# Validation of the post sleep questionnaire for assessing subjects with restless legs syndrome: results from two double-blind, multicenter, placebo-controlled clinical trials

**DOI:** 10.1186/1471-2377-11-48

**Published:** 2011-04-28

**Authors:** Daniel M Canafax, Abhijeet Bhanegaonkar, Murtuza Bharmal, Michael Calloway

**Affiliations:** 1 XenoPort, Inc., Santa Clara, CA, USA; 2 West Virginia University School of Pharmacy, Morgantown, WV, USA; 3 Current Address: GlaxoSmithKline R&D China, Shanghai, China; 4 Previous Address: Quintiles, Rockville, MD, USA; 5 GlaxoSmithKline, Research Triangle Park, NC, USA

## Abstract

**Background:**

Because of the subjective nature of Restless Legs Syndrome (RLS) symptoms and the impact of these symptoms on sleep, patient-reported outcomes (PROs) play a prominent role as study endpoints in clinical trials investigating RLS treatments. The objective of this study was to validate a new measure, the Post Sleep Questionnaire (PSQ), to assess sleep dysfunction in subjects with moderate-to-severe RLS symptoms.

**Methods:**

Pooled data were analyzed from two 12-week, randomized, placebo-controlled trials of gabapentin enacarbil (N = 540). At baseline and Week 12, subjects completed the PSQ and other validated health surveys: IRLS Rating Scale, Clinical Global Impression of Improvement (CGI-I), Profile of Mood States (POMS), Medical Outcomes Study Scale-Sleep (MOS-Sleep), and RLS-Quality of Life (RLSQoL). Pooled data were used *post hoc *to examine the convergent, divergent, known-group validity and the responsiveness of the PSQ.

**Results:**

Convergent validity was demonstrated by significant correlations between baseline PSQ items and total scores of IRLS, POMS, RLSQoL, and the MOS-Sleep Scale (p ≤ 0.007 each). Divergent validity was demonstrated through the lack of significant correlations between PSQ items and demographic characteristics. Correlations (p < 0.0001) between RLS severity groups and PSQ items demonstrated known-group validity. Mean changes in investigator- and subject-rated CGI-I scores for each PSQ item (p < 0.0001) demonstrated the PSQ's responsiveness to patient change as reported by their care provider.

**Conclusions:**

Although these analyses were potentially limited by the use of clinical trial data and not prospective data from a study conducted solely for validation purposes, the PSQ demonstrated robust psychometric properties and is a valid instrument for assessing sleep and sleep improvements in subjects with moderate-to-severe RLS symptoms.

**Trial Registration:**

This study analyzed data from two registered trials,  NCT00298623 and  NCT00365352.

## Background

Restless Legs Syndrome (RLS) is a sensorimotor disorder that can substantially disrupt sleep. It affects 5-10% of the general population [1], predominantly women [1,2], and has been characterized as chronic in individuals with moderate-to-severe symptoms requiring treatment [3]. The criteria that define RLS include an urge to move the legs due to unpleasant or uncomfortable sensations during periods of rest, the relief of symptoms through leg movements, and symptoms that follow a distinct circadian pattern [3]. The onset of RLS symptoms typically begin in the late evening and may persist through the night time hours. Thus, the primary morbidity in RLS is sleep disruption, the major reason patients cite for consulting their physicians [2,3].

Sleep-related problems, such as trouble initiating or maintaining sleep and experiencing disturbed, non-restful or non-refreshing sleep, are more prevalent in patients with RLS than in those without RLS [4]. Nearly 90% of patients with RLS experience at least 1 sleep-associated symptom and over 40% of patients identified sleep-associated issues as the most bothersome RLS-related symptom [5]. Nearly 70% of patients with twice weekly RLS symptoms needed more than 30 minutes to fall asleep and 60% had more than 3 awakenings per night. On average, patients with severe RLS symptoms sleep only 4 to 5 hours per night [5].

Because of the subjective nature of RLS symptoms and the impact of these symptoms on sleep, patient-reported outcomes (PROs) play a prominent role as study endpoints in clinical trials investigating RLS treatments. However, for PROs to be credible, they must be subjected to a rigorous inspection of their measurement properties using measurement theory and standard psychometric assessments. While there were several pre-existing sleep instruments to choose from (i.e., MOS Sleep Questionnaire) most were limited in their use in RLS research because they were not disease specific, or were more difficult to administer within a clinical trial setting (i.e., clinician-administered). The hope was to be more responsive to the Food and Drug Administration's (FDA) recently emphasized expectations around patient reported outcome measures and the need to demonstrate the validity and reliability of such scales when used to measure endpoints in clinical trials [6].

The Post Sleep Questionnaire (PSQ) is a, 5-item PRO instrument designed to evaluate sleep disturbance in subjects with RLS. This study was a *post-hoc *evaluation of the validity of the PSQ, Version 2, using data from 2 well-controlled clinical trials in subjects with moderate-to-severe RLS [7,8].

## Methods

### Data source

This study used pooled data from two 12-week, multicenter, randomized, double-blind, placebo-controlled clinical trials (XenoPort, Inc. protocols XP052 [ClinicalTrials.gov Identifier NCT00298623] and XP053 [ClinicalTrials.gov Identifier NCT00365352]) that evaluated the efficacy and tolerability of gabapentin enacarbil (GEn) for the treatment of moderate-to-severe primary RLS [7,8]. The co-primary endpoints for the 1200 mg and placebo groups in both studies were the reduction in total IRLS score from baseline to Week 12 on the International Restless Legs Scale (IRLS) [9] and the proportion of responders rated by investigators as "very much improved" or "much improved" on the Clinical Global Impression-Improvement [CGI-I] scale) [10]. The secondary endpoints evaluated subject-rated improvements in sleep, mood, and quality of life.

The studies were of similar design. Subjects were randomly assigned for 12 weeks of treatment to GEn 1200 mg or matching placebo (XP052, XP053), with study XP053 also examining a third treatment group, GEn 600 mg, as a secondary endpoint compared with placebo. These studies demonstrated statistically significant efficacy with both GEn doses compared with placebo for reduction of RLS symptoms and improvements in sleep, mood, and quality of life [7,8].

### Study population

Study methodologies have been published elsewhere [7,8]. Briefly, eligible subjects were ≥18 years of age, diagnosed with primary RLS, having RLS symptoms ≥15 nights of the month prior to study enrollement and for ≥4 of 7 consecutive nights in the week prior to their baseline assessment, and had a total RLS severity score of ≥15 (i.e., moderate-to-severe severity). If subjects were receiving RLS treatment at screening, then they must have had a symptom frequency of ≥15 nights per month prior to treatment initiation. A 2-week washout period prior to the baseline assessments was required for dopamine agonists, gabapentin, opioids, and benzodiazepines.

Subjects with non-RLS-related sleep disorders (e.g., sleep apnea), a history of RLS symptom augmentation or early-morning rebound with previous dopamine-agonist treatment, neurological disease or movement disorders other than RLS (e.g., diabetic neuropathy, Parkinson's disease, multiple sclerosis, dyskinesias, and dystonias), or other medical conditions that could confound study results were excluded.

### PSQ

**Figure 1:**
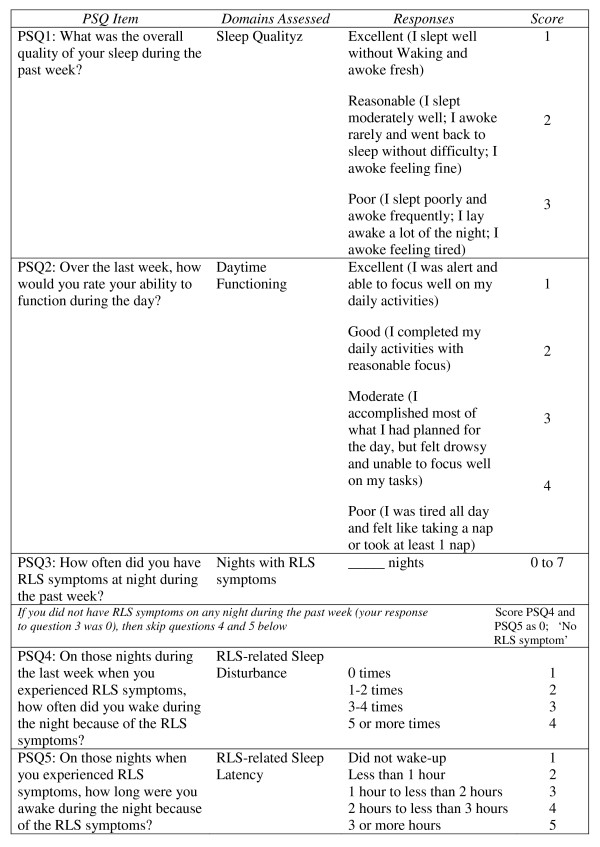
**The 5-item PSQ with assessed domains, possible responses, and item scores**. Higher scores indicate greater sleep difficulties associated with RLS.

Figure 1 displays the 5 items comprising the PSQ, the sleep domains corresponding to each question, the associated response categories, and the scoring for each. The PSQ was developed to evaluate sleep disturbance in subjects with primary RLS over the past weeks using 4 Likert-type scale questions and 1 quantitative, open-ended question. The PSQ assesses several single-item sleep domains, including overall sleep quality, overall daytime functioning, frequency of night time RLS symptoms, and RLS-related sleep disturbances and latency. Higher PSQ scores indicate worse sleep.

### Validated sleep instruments

Seven PRO instruments were completed as part of the clinical trials assessments. Five of the instruments were previously validated and were used in the present study to validate the PSQ: 1) IRLS Rating Scale comprises 10 questions that assess symptom severity and frequency on a 40-point scale (0 = no symptom; 40 = very severe symptoms); subjects were also classified into RLS symptom severity categories using the IRLS total score (mild = 0-10, moderate = 11-20, severe = 21-30, and very severe ≥31) [11]; 2) investigator- and subject-rated CGI-I, evaluates improvements in any disease on a 7-point scale (1 = "very much improved"; 7 = "very much worse") [10]; 3) Profile of Mood States (POMS) is a 30-item scale that assesses mood in 6 domains and 1 total overall mood score, with higher scores indicating a more negative mood state [12]; 4) Medical Outcomes Study (MOS) Scale-Sleep assesses sleep constructs, including sleep initiation, maintenance, perceived adequacy, somnolence, respiratory impairments, and regularity in subjects with health comorbidities, such as RLS [13-16]; and 5) RLS Quality of Life Questionnaire (RLSQoL), a 100-point scale that assesses the direct impact of RLS on the daily life, emotional well-being, social life, and work-life of the subject, with lower scores indicating better quality of life [17,18]. The remaining 2 instruments were diaries that provided important subject response information: 1) the 24-hour RLS Record assesses the frequency of RLS symptoms in half-hour increments during a 24-hour period and 2) the 7-Day RLS Symptom Diary assesses the frequency of nightly symptoms over a 1-week period.

### Analyses

Analyses were conducted on pooled data irrespective of treatment assignment, as baseline assessments were used to generate the psychometric indices. Data from 540 subjects were included in these analyses: 220 from XP052 and 320 from XP053. As shown in Table 1, there were no major differences between the 2 study populations at baseline in terms of age, gender, ethnicity, or disease severity.

**Table 1:** Baseline demographic and characteristics of enrolled subjects from two clinical trials conducted in subjects with primary RLS

Characteristic	XP052 (n = 220)	XP053 (n = 320)	All (n = 540)^2^
Age			
Mean (SD)	51.5 (12.75)	49.3 (12.55)	50.2 (12.66)
Female (n, %)	131 (59.5%)	189 (59.1%)	320 (59.3%)
Race (n, %)			
White or Caucasian	212 (96.4)	303 (94.7)	515 (95.4)
Black or African-American	5 (2.3)	5 (1.6)	10 (1.9)
Other	3 (1.3)	12 (3.7)	15 (2.7)
Ethnicity (n, %)			
Hispanic or Latino	8 (3.6)	25 (7.8)	33 (6.1)
Non-Hispanic or Latino	212 (96.4)	295 (92.2)	507 (93.9)
Treatment (n, %)			
1200 mg	113 (51.4)	111 (34.7)	224 (41.5)
600 mg	0	113 (35.3)	113 (20.9)
Placebo	107 (48.6)	96 (30.0)	203 (37.6)
RLS Severity (n, %)^1^			
Mild	0	0	0
Moderate	82 (37.3)	102 (31.9)	184 (34.1)
Severe	121 (55.0)	193 (60.3)	314 (58.1)
Very severe	17 (7.7)	25 (7.8)	42 (7.8)

RLS, Restless Legs Syndrome; SD, standard deviation.

^1^RLS Disease Severity based on International Restless Legs Rating Scale Total Score, where mild = 0-10, moderate = 11-20, severe = 21-30, and very severe >30.

**^2^**In the original clinical trials; there were a total of 544 subjects. In this study we used the Post Sleep Questionnaire (PSQ) validation sample, which includes only those who completed the PSQ at Baseline (N = 540) and Week 12 (N = 451).

The following psychometric properties were assessed.

### Convergent validity

Convergent validity, the extent of agreement between 2 scales that measure similar domains or criteria that are linked with the domain of interest, was evaluated using the nonparametric Spearman-rank correlation coefficient. Convergent validity is accepted when the correlation estimates are in the correct theoretical direction [19] and are statistically significant. If correlation coefficients are statistically significant, Cohen [20] describes correlations of 0.1 as small, 0.3 as medium, and ≥0.5 as large convergence. Convergent validity for the PSQ was determined by correlating the PSQ item scores with the total scores from the IRLS, RLSQoL, and POMS, and the 4 individual domain scores of the MOS-Sleep scale.

Individual PSQ item scores were also correlated with similar items from daily diaries that captured RLS-related symptoms and sleep: PSQ Item 3 (nights with RLS symptoms) score and nights with RLS symptoms score from the 7-Day RLS Symptom Diary, PSQ Item 4 (RLS-related sleep disturbance) with number of RLS-related awakenings score from the 24-Hour RLS Symptom Diary, and PSQ Item 5 (RLS-related sleep latency) with the RLS-related sleep latency score from the 24-Hour RLS Record.

### Divergent validity

An instrument demonstrates divergent validity when it shows a lack of statistical association with other instruments or related variables (e.g., demographic characteristics) unrelated to the focus instrument. This study was limited in the choice of instruments to assess divergent validity because it utilized only the instruments included in the clinical trials [7,8] that were used to measure the effects of RLS treatment. To demonstrate divergent validity of the PSQ, we assessed correlations between the baseline PSQ item scores and three demographic variables: ethnicity, race, and gender. Because there was no theoretical basis to expect a relationship between sleep problems and these demographic variables, a lack of correlation would demonstrate that subjects with different demographic characteristics do not interpret the PSQ items differently. Cramer's V statistic was used to assess their associations [21], because this involved correlating a nominally scaled variable (i.e., 0 = Male, 1 = Female) with continuous variables. Cramer's V estimates range from 0 to 1, with low scores indicating poor association and demonstrating divergent validity.

### Known-group validity

Known-group validity, or predictive validity, is the ability of a scale or scale items to statistically discriminate respondents in ways expected (predicted) as a result of independent assessments. The known groups in the current study were based on RLS symptom severity, as determined by the IRLS total score. Sleep qualities measured by the PSQ are expected to be most pronounced for persons with severe symptoms. Baseline PSQ scores were compared across severity groups using the Kruskal-Wallis test [22].

### Responsiveness

Responsiveness means that the PSQ item scores will change in direction and magnitude with changes experienced by the responders. In this case, responsiveness was demonstrated if relationships between changes in the investigator- and subject-rated CGI-I assessments and changes in PSQ scores over time were in the same direction and statistically significant [15]. Change from baseline in individual PSQ items were compared with subject symptom changes as measured by the CGI-I using the Kruskal-Wallis test. Among all subjects in the clinical trials [7,8], only a few experienced worsening of symptoms over time; as a result, the responsiveness analysis was conducted by re-categorizing subjects reporting 'minimally worse', 'much worse' and 'very much worse' into 1 category of 'worse'. Guyatt's statistic, a measure of test-retest reliability or responsiveness to change, which is the ratio of the mean change for each category divided by the standard deviation for the no change group, was estimated. An instrument with an absolute value of ≥0.20 for Guyatt's statistic has acceptable responsiveness and a value of ≥1.00 is considered highly responsive to change [23,24].

## Results

### Subject characteristics

Of 544 total subjects, 540 and 451 completed the PSQ at Baseline and Week 12, respectively, and were included in the analysis. Table 1 shows that the baseline mean age of subjects was approximately 50 years, most subjects were non-Hispanic, white, and female. About 92% had moderate or severe RLS symptoms and 8% had very severe symptoms.

### PSQ results

The mean scores for PSQ items indicate that most subjects improved over the 12-week study, and most of the improvements occurred by Week 4 (Table 2). For sleep quality, the percentage of subjects reporting their sleep quality as "excellent" increased by approximately 20% by Week 12. Daytime functioning showed similar positive gains, with 4.5 times as many subjects reporting their daytime functioning as "excellent" at Week 12 compared with baseline. Similar improvements in frequency of nighttime RLS symptoms, RLS-related sleep disturbances and sleep latency over the 12 weeks were observed.

**Table 2:** Subject responses and mean scores for PSQ items

Overall sleep quality				
Excellent, n (%)	3 (0.6)	98 (20.1)	78 (16.6)	91 (20.2)
Reasonable, n (%)	209 (38.7)	294 (60.2)	293 (62.2)	279 (61.9)
Poor, n (%)	328 (60.7)	96 (19.7)	100 (21.2)	81 (18.0)
Mean PSQ score (SD)	2.602 (0.501)	1.996 (0.631)	2.047 (0.614)	1.978 (0.618)
Daytime functioning				
Excellent, n (%)	35 (6.5)	146 (29.9)	163 (34.6)	159 (35.3)
Good, n (%)	240 (44.4)	238 (48.8)	222 (47.1)	220 (48.8)
Moderate, n (%)	230 (42.6)	95 (19.5)	80 (17.0)	63 (14.0)
Poor, n (%)	35 (6.5)	9 (1.8)	6 (1.3)	9 (2.0)
Mean PSQ score (SD)	2.491 (0.714)	1.932 (0.751)	1.849 (0.738)	1.827 (0.737)

Frequency of nighttime RLS symptoms				
0	0	83 (17.0)	99 (21.0)	109 (24.2)
1	9 (1.7)	144 (29.5)	133 (28.2)	126 (27.9)
2	46 (8.5)	107 (21.9)	103 (21.9)	77 (17.1)
3	187 (34.6)	61 (12.5)	57 (12.1)	67 (14.9)
4	298 (55.2)	93 (19.1)	79 (16.8)	72 (16.0)
Mean PSQ score (SD)	3.433 (0.719)	1.871 (1.360)	1.754 (1.363)	1.705 (1.397)

Frequency of RLS-related sleep disturbance				
No RLS symptoms	0	83 (17.0)	99 (21.0)	109 (24.2)
0 times	59 (10.9)	135 (27.7)	111 (23.6)	115 (25.5)
1-2 times	262 (48.5)	203 (41.6)	205 (43.5)	184 (40.8)
3-4 times	163 (30.2)	49 (10.0)	48 (10.2)	36 (8.0)
≥5 times	56 (10.4)	18 (3.7)	8 (1.7)	7 (1.6)
Mean PSQ score (SD)	2.400 (0.817)	1.557 (1.006)	1.480 (0.989)	1.373 (0.986)

RLS-related sleep latency (time awake)				
No RLS symptoms	0	83 (17.0)	99 (21.0)	109 (24.2)
Did not wake-up	59 (10.9)	135 (27.7)	111 (23.6)	115 (25.5)
<1 hour	240 (44.4)	176 (36.1)	180 (38.2)	160 (35.5)
1-<2 hours	133 (24.6)	67 (13.7)	57 (12.1)	45 (10.0)
2-<3 hours	74 (13.7)	12 (2.5)	18 (3.8)	12 (2.7)
≥3 hours	34 (6.3)	15 (3.1)	6 (1.3)	10 (2.2)
Mean PSQ score (SD)	2.600 (1.055)	1.662 (1.165)	1.580 (1.136)	1.481 (1.167)

PSQ, Post Sleep Questionnaire; RLS, Restless Legs Syndrome; SD, standard deviation.

^1^All responses were based on experiences during the previous week.

Higher PSQ scores correspond to worse symptoms.

### Convergent validity

Table 3 shows the Spearman rank correlation coefficients and the probability of each PSQ item based on the total scores of the IRLS, RLSQoL, and POMS. All correlations between the IRLS total score and each PSQ item were statistically significant, ranging from 0.25 to 0.49 (p > 0.0001, each). The strength of the associations showed moderate to moderately high convergence. The overall POMS total score had statistically significant correlations with the PSQ items, except for the item "nights with RLS symptoms". The correlations ranged from 0.04 to 0.48. All but 1 of the correlations were small in size; daytime functioning was moderately high. For the individual POMS items, 22 of 35 correlations were statistically significant (p < 0.05 each; data not shown). The correlations between the RLSQoL total score and the PSQ items were also statistically significant. The correlation coefficients ranged from -0.12 to -0.57 (p < 0.01 each). The convergence was large for daytime functioning, moderate for sleep quality, and small for all others.

**Table 3:** Convergent validity: PSQ item correlations at baseline with IRLS, POMS, and RLSQoL total scores

PSQ item	IRLS total score (n = 540)	POMS overall mood score (n = 539)	RLSQoL total score (n = 540)
	
	Correlation *(*p-value)	Correlation (p-value)	Correlation (p-value)
Sleep quality	0.4635 (<0.0001)	0.2319 (<0.0001)	-0.2986 (<0.0001)

Daytime functioning	0.4890 (<0.0001)	0.4818 (<0.0001)	-0.5663 (<0.0001)

Nights with RLS symptoms	0.2509 (<0.0001)	0.0388 (0.3690)	-0.1154 (0.0073)

RLS-related sleep disturbance	0.3186 (<0.0001)	0.1174 (0.0063)	-0.2371 (<0.0001)

RLS-related sleep latency	0.3725 (<0.0001)	0.1205 (0.0051)	-0.2870 (<0.0001)

IRLS, International Restless Legs Rating Scale; POMS, Profile of Mood States; PSQ, Post Sleep Questionnaire; RLSQoL, Restless Legs Syndrome Quality of Life Questionnaire.

Spearman Correlation coefficients.

Higher PSQ scores correspond to worse RLS symptoms.

Higher IRLS Total scores correspond to higher severity of restless leg syndrome symptoms.

Higher POMS scores correspond to increased negative mood affect.

Higher RLS Quality of life scores correspond to better quality of life.

The correlations between the MOS-Sleep scale domains and the PSQ items are provided in Table 4. The correlation coefficients ranged from -0.46 to 0.49; 18 of 20 correlations were statistically significant (p < 0.02). Of the 20 correlations, 9 had moderate to moderately large coefficients. Moderately high convergence was seen between the PSQ sleep quality item and the MOS sleep disturbance domain (higher sleep disturbance, lower sleep quality) and sleep adequacy (higher sleep quality, higher sleep adequacy), between PSQ daytime functioning and MOS daytime somnolence domain (worse functioning, worse somnolence), between PSQ sleep latency and MOS sleep disturbance domain (higher sleep latency, higher sleep disturbance). The correlations of the PSQ item nights with RLS symptoms were statistically significant with MOS sleep domains, but showed small convergence with MOS sleep disturbance, adequacy and quantity domains.

**Table 4:** Convergent validity: PSQ items and MOS Sleep domains at baseline

PSQ item	MOS Sleep Domain Scores			
	
	Daytime somnolence n = 540	Sleep disturbance n = 540	Sleep adequacy n = 540	Sleep quantity n = 539
	
	Correlation (p-value)	Correlation (p-value)	Correlation (p-value)	Correlation (p-value)
Sleep quality	0.1856 (<0.0001)	0.4878 (<0.0001)	-0.4635 (<0.0001)	-0.3827 (<0.0001)

Daytime functioning	0.4290 (<0.0001)	0.3027 (<0.0001)	-0.4084 (<0.0001)	-0.2287 (<0.0001)

Nights with RLS symptoms	0.0458 (0.2881)	0.1025 (0.0172)	-0.1537 (0.0003)	-0.1207 (0.0050)

RLS-related sleep disturbance	0.1369 (0.0014)	0.3158 (<0.0001)	-0.2188 (<0.0001)	-0.2742 (<0.0001)

RLS-related sleep latency	0.0633 (0.1420)	0.4290 (<0.0001)	-0.2083 (<0.0001)	-0.3798 (<0.0001)

MOS, Medical Outcomes Study; PSQ, Post Sleep Questionnaire; RLS, restless legs syndrome.

Spearman Correlation coefficients.

Higher PSQ scores correspond to worse symptoms.

Higher MOS Daytime Somnolence scores and MOS Sleep Disturbance scores correspond to worse symptoms.

Higher MOS Sleep Adequacy scores and MOS Sleep Quantity scores correspond to better symptoms.

The 7-day RLS Symptom Diary significantly correlated with the PSQ item #3 nights with RLS symptoms (r = 0.47; p < 0.0001; data not shown). The correlation between the 24-hour RLS Symptom Record Diary and the PSQ items RLS-related sleep disturbance and RLS-related sleep latency were 0.17 and 0.27, respectively (p < 0.0001, both).

### Divergent validity

None of the correlation estimates from Cramer's V for any PSQ item baseline score across the 3 demographic variables (i.e., race, gender and ethnicity) were larger than 0.13 (data not shown) and none approached statistical significance, thereby demonstrating divergent validity. For race, the correlation estimates ranged from 0.05 for sleep latency to 0.13 for sleep disturbance. The respective estimates for ethnicity ranged from 0.02 to 0.07, and for gender, the range was 0.05 to 0.12.

### Known-group validity

Positive and statistically significant differences (p < 0.0001, each) were found between baseline PSQ scores and the RLS severity groups moderate, severe, and very severe (Table 5). The estimates were linear across the severity groups with very severe RLS subjects having worse PSQ scores than subjects with severe RLS and subjects with severe RLS having worse PSQ scores than subjects with moderate RLS.

**Table 5:** Known-group validity PSQ items and RLS disease severity as assessed by IRLS total score at baseline

PSQ item score, mean (SD)	RLS disease severity			p-value
	
	Moderate n = 184	Severe n = 314	Very Severe n = 42	
Sleep quality	2.342 (0.509)	2.710 (0.454)	2.929 (0.261)	<0.0001

Daytime functioning	2.125 (0.619)	2.611 (0.651)	3.190 (0.740)	<0.0001

Nights with RLS symptoms	3.228 (0.777)	3.535 (0.664)	3.571 (0.668)	<0.0001

RLS-related sleep disturbance	2.136 (0.774)	2.465 (0.792)	3.071 (0.712)	<0.0001

RLS-related sleep latency	2.190 (0.869)	2.720 (1.035)	3.500 (1.174)	<0.0001

IRLS, International Restless Legs Rating Scale; PSQ, Post Sleep Questionnaire; RLS, Restless Legs Syndrome; SD, standard deviation.

Higher PSQ scores correspond to worse symptoms.

p-value is based on Kruskal-Wallis test.

RLS Disease Severity based on IRLS Total Score, where mild = 0-10, moderate = 11-20, severe = 21-30, and very severe >30.

### Responsiveness

The mean change in PSQ scores from baseline to Week 12 indicate that improvements in sleep were achieved and scores were significantly improved across each of the investigator- and subject-rated CGI-I categories (Tables 6 and 7, respectively). The largest change was among those subjects judged by the clinician as being "very much improved" followed by "much improved", "minimally improved", "no change", and finally "worse." The magnitude of the effect size for change in PSQ scores from baseline also followed the above linear order (data not shown). The magnitude of Guyatt's statistic was moderate to large, ranging from 0.00 to 3.46 among improved subjects. Of the 50 coefficients, 7 did not meet the acceptable cutpoint of 0.20 or greater and all but 1 of the 7 was in the "worse" category. Additionally, 11 of the 50 coefficients were >1, indicating a highly responsive measure.

**Table 6:** Responsiveness: mean change from baseline in PSQ item scores and Guyatt's statistic by investigator-rated CGI-I at Week 12

	Investigator-rated CGI-I					
		
PSQ domains	Very Much Improved n = 173	Much Improved n = 126	Minimally Improved n = 64	No Change n = 73	Worsen n = 7	p-value
Sleep quality						
Mean delta (SD)	-0.92 (0.74)	-0.67 (0.63)	-0.34 (0.60)	-0.14 (0.56)	0.14 (0.38)	<0.0001
Guyatt's statistic	1.64	1.19	0.61	0.24	0.25	

Daytime functioning						
Mean delta (SD)	-1.03 (0.91)	-0.62 (0.77)	-0.34 (0.76)	-0.15 (0.78)	0.14 (0.38)	<0.0001
Guyatt's statistic	1.33	0.80	0.44	0.19	0.18	

Nights with RLS symptoms						
Mean delta (SD)	-2.71 (1.03)	-1.75 (1.20)	-0.97 (1.10)	-0.26 (0.94)	0.57 (0.53)	<0.0001
Guyatt's statistic	2.87	1.86	1.03	0.28	0.61	

RLS-related sleep disturbance						
Mean delta (SD)	-1.72 (1.14)	-0.80 (0.96)	-0.58 (0.83)	-0.25 (0.95)	-1.14 (1.07)	<0.0001
Guyatt's statistic	1.81	0.84	0.61	0.26	1.20	

RLS-related sleep latency						
Mean delta (SD)	-1.82 (1.47)	-0.80 (1.18)	-0.69 (1.40)	-0.19 (1.09)	-1.29 (0.95)	<0.0001
Guyatt's statistic	1.67	0.74	0.63	0.18	1.18	

CGI-I, Clinical Global Impression-Improvement, Investigator-rated; PSQ = Post Sleep Questionnaire; SD, standard deviation.

Higher PSQ scores correspond to worse symptoms.

Mean delta is change from baseline to Week 12.

p-value is based on Kruskal-Wallis test.

Guyatt's statistic is the ratio of the mean change score for each category of subjects divided by the standard deviation for the no change group.

**Table 7:** Responsiveness: mean change from baseline in PSQ item scores, effect size, and Guyatt's statistic by subject-rated CGI-I at Week 12

	Subject-rated Clinical Global Impression of Change					
PSQ domains	Very Much Improved n = 175	Much Improved n = 130	Minimally Improved n = 61	No Change n = 65	Worse n = 15	p-value
Sleep quality						
Mean delta (SD)	-0.92 (0.77)	-0.68 (0.60)	-0.31 (0.59)	-0.14 (0.56)	0.00 (0.38)	<0.0001
Guyatt's statistic	1.66	1.23	0.56	0.25	0.00	
Daytime functioning						
Mean delta (SD)	-1.01 (0.92)	-0.68 (0.77)	-0.33 (0.60)	-0.05 (0.80)	-0.13 (0.74)	<0.0001
Guyatt's statistic	1.27	0.85	0.41	0.06	0.17	
Nights with RLS symptoms						
Mean selta (SD)	-2.74 (0.99)	-1.70 (1.22)	-0.93 (1.06)	-0.25 (0.79)	0.00 (1.41)	<0.0001
Guyatt's statistic	3.46	2.15	1.18	0.31	0.00	
RLS-related sleep disturbance						
Mean delta (SD)	-1.67 (1.15)	-0.89 (1.01)	-0.48 (0.74)	-0.32 (0.94)	-0.47 (1.19)	<0.0001
Guyatt's statistic	1.78	0.95	0.51	0.34	0.50	
RLS-related sleep latency						
Mean delta (SD)	-1.82 (1.41)	-0.86 (1.35)	-0.38 (1.20)	-0.32 (1.00)	-0.73 (1.44)	<0.0001
Guyatt's statistic	1.82	0.86	0.38	0.32	0.73	

CGI-I, Clinical Global Impression-Improvement, subject-rated; SD = Standard Deviation

Mean delta is change from baseline to Week 12

p-value is based on Kruskal-Wallis test

Effect size based on Cohen's d is the difference in the mean score/pooled standard deviation.

Guyatt's statistic is the ratio of the mean change score for each category of subjects divided by the standard deviation for the no change group.

## Discussion

This *post hoc *psychometric evaluation of an investigator-developed PRO instrument used data from 2 randomized, double-blind, placebo-controlled trials conducted in subjects with moderate-to-severe primary RLS to examine the validity of the PSQ in assessing RLS-related sleep disturbance and treatment improvements. The parent studies indicated that the PSQ scores showed improved sleep in subjects with RLS symptoms who received active RLS treatment compared with placebo [7,8].

To verify the validity of the PSQ, several measurement properties were evaluated. The PSQ's convergent validity was estimated using correlations between the overall RLS symptom impact score, as assessed with the IRLS, and the individual PSQ items. These correlation estimates were all statistically significant and positive (i.e., worse symptom impact with worse sleep status). All PSQ items scores had moderate convergence with overall RLS symptom impact, except for the PSQ item "nights with RLS symptoms", which had a lower convergence.

Convergent validity was also assessed using the MOS Sleep Scale. Nearly half of the correlations with the MOS-Sleep Scale sleep domains showed moderate to moderately high convergence. Two PSQ items, *nights with RLS symptoms *and *RLS-related sleep latency*, showed little convergence with the MOS daytime somnolence domain. The PSQ sleep quality domain had sufficient convergence with the MOS sleep disturbance, sleep adequacy and sleep quantity domains. The PSQ daytime functioning item had sufficient convergence with all the MOS domains. The PSQ item, RLS-related sleep latency, had sufficient convergence with MOS sleep domains, except daytime somnolence. Taken together, these data suggest that the PSQ items had adequate convergence with the most pertinent MOS sleep domains.

Validity can also be shown via divergence with scales intended to measure other concepts. We found no strong associations across the 3 demographic variables of race, ethnicity and gender with the PSQ items. While demographics are not traditionally used for assessing divergent validity, we were limited to using the data collected in the trials. Given that, these findings clearly and robustly demonstrate that the PSQ results do not include systematic measurement errors that could be associated with personal attributes.

The PSQ results showed a consistent and robust ability to discriminate between RLS severity groups in a known-group validity analysis. All estimates were linear across the severity groups with increasingly worse sleep outcomes found in progressively worse symptom groups.

The PSQ was found to provide reliable results; for example, the PSQ was responsive to treatment differences over 12 weeks, and the findings were consistent regardless of whether the investigator or the subject rated the improvement, as indicated by the clinician and the patient rated CGI-Is. Further, the changes were seen regardless of treatment assignment, indicating that the PSQ is sensitive to small clinical changes over the course of treatment. Guyatt's statistic, an indication of responsiveness to change, was acceptably strong in all responder categories that indicated change and comparatively weaker in groups that indicated no change or worse change.

The *post hoc *nature of this study posed some challenges. Analyses were confined to the instruments collected as part of the clinical trials and were not included for the purposes of conducting a validation study. Thus, some aspects of the instruments used to validate the PSQ may not have been ideal. For example, items on some instruments are not directly related to sleep or are confounded by sleep. Finally, the recall periods varied among the instruments and some instruments were not specifically designed for RLS.

## Conclusions

The PSQ demonstrated acceptable measurement properties of convergent validity, divergent validity, and known-group validity. PSQ scores were also reliable in terms of being responsive to change in symptoms. The PSQ is, therefore, a psychometrically valid instrument for assessing sleep among RLS subjects in clinical trial settings.

## Competing interests

**DMC **is an employee of XenoPort, Inc. where the RLS clinical studies were designed and conducted. **AJB **worked on this project as a graduate intern from West Virginia University at GlaxoSmithKline, Research Triangle Park, NC. **MFB **was a full-time employee of Quintiles, Inc., (the company that GlaxoSmithKline sponsored to conduct the psychometric research) at the time this study was conducted. **MOC **is a full-time employee of GlaxoSmithKline, Research Triangle Park, NC.

## Authors' contributions

All the authors contributed to the manuscript and have read and approved the final version.

DMC: project management, study design, execution, medical monitoring, analysis, and primary author. AJB: psychometric design, contributing author. MFB: study design, psychometric design and analysis, project management, contributing author. MOC: study design, psychometric design and data analysis, project management, contributing author.

## Pre-publication history

The pre-publication history for this paper can be accessed here:

http://www.biomedcentral.com/1471-2377/11/48/prepub
